# Enhanced Blood Suspensibility and Laser-Activated Tumor-specific Drug Release of Theranostic Mesoporous Silica Nanoparticles by Functionalizing with Erythrocyte Membranes: Erratum

**DOI:** 10.7150/thno.42848

**Published:** 2020-01-18

**Authors:** Jinghan Su, Huiping Sun, Qingshuo Meng, Pengcheng Zhang, Qi Yin, Yaping Li

**Affiliations:** 1 State Key Laboratory of Drug Research & Center of Pharmaceutics, Shanghai Institute of Materia Medica, Chinese Academy of Sciences, 501 Haike Road, Shanghai 201203, China;; 2 University of Chinese Academy of Sciences, Beijing 100049, China;; 3 School of Pharmacy, Shenyang Pharmaceutical University, Shenyang, 110016, China.

In the initially published version of this article, the “Heart” image of the RMSNs+Laser group in Figure 7 is wrong. The correct Figure 7 is as follow:

The corrections made in this erratum do not affect the original conclusions. The authors apologize for any inconvenience or misunderstanding that this error may have caused.

## Figures and Tables

**Figure 7:**
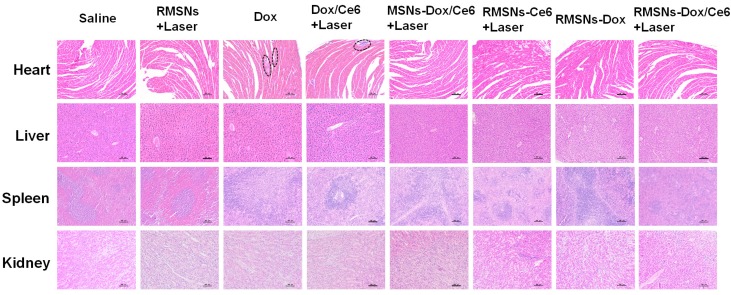
H&E staining of heart, liver, spleen and kidney (100×) at the end of the antitumor inhibition test. The black circles indicated the inflammation in the heart of the Dox-treated mice. Scale bar = 100 μm.
